# Deciphering Microbial Shifts in the Gut and Lung Microbiomes of COVID-19 Patients

**DOI:** 10.3390/microorganisms12061058

**Published:** 2024-05-24

**Authors:** Vaidehi Pusadkar, Anirudh Mazumder, Abhijay Azad, Deepti Patil, Rajeev K. Azad

**Affiliations:** 1 Department of Biological Sciences and BioDiscovery Institute, University of North Texas, Denton, TX 76203, USA; vaidehipusadkar@my.unt.edu; 2 Texas Academy of Mathematics and Science, University of North Texas, Denton, TX 76203, USA

**Keywords:** metagenomics, taxonomic profiling, microbiome, COVID-19

## Abstract

COVID-19, caused by SARS-CoV-2, results in respiratory and cardiopulmonary infections. There is an urgent need to understand not just the pathogenic mechanisms of this disease but also its impact on the physiology of different organs and microbiomes. Multiple studies have reported the effects of COVID-19 on the gastrointestinal microbiota, such as promoting dysbiosis (imbalances in the microbiome) following the disease’s progression. Deconstructing the dynamic changes in microbiome composition that are specifically correlated with COVID-19 patients remains a challenge. Motivated by this problem, we implemented a biomarker discovery pipeline to identify candidate microbes specific to COVID-19. This involved a meta-analysis of large-scale COVID-19 metagenomic data to decipher the impact of COVID-19 on the human gut and respiratory microbiomes. Metagenomic studies of the gut and respiratory microbiomes of COVID-19 patients and of microbiomes from other respiratory diseases with symptoms similar to or overlapping with COVID-19 revealed 1169 and 131 differentially abundant microbes in the human gut and respiratory microbiomes, respectively, that uniquely associate with COVID-19. Furthermore, by utilizing machine learning models (LASSO and XGBoost), we demonstrated the power of microbial features in separating COVID-19 samples from metagenomic samples representing other respiratory diseases and controls (healthy individuals), achieving an overall accuracy of over 80%. Overall, our study provides insights into the microbiome shifts occurring in COVID-19 patients, shining a new light on the compositional changes.

## 1. Introduction

Advances in next-generation sequencing (NGS) technologies have revolutionized our understanding of disease pathogenesis across multiple organs and their interactions with the microbiome. Metagenomic analysis approaches have shown that the dysbiosis of specific opportunistic pathogens and beneficial microbial communities in human diseases significantly influence their severity [1,2,3]. COVID-19 is a respiratory infection that, in most cases, is mild or moderate but can lead to severe symptoms, particularly in people with comorbidities [4]. The causative agent, SARS-CoV-2, a single-stranded, positive-sense RNA virus of the Betacoronavirus genus, triggered the COVID-19 pandemic with profound public health and socioeconomic consequences worldwide. The cumulative total of COVID-19 cases reported to the WHO exceeds 774M worldwide as of now (https://data.who.int/dashboards/covid19/cases?n=c, accessed on 31 March 2024). In addition to respiratory symptoms such as cough, shortness of breath, fever, fatigue, and abnormal chest X-rays [5], COVID-19 patients also exhibit gastrointestinal manifestations such as diarrhea, nausea or vomiting, anorexia, and abdominal pain [6,7].

Numerous studies have highlighted the correlation between COVID-19 and broad alterations in microbial communities, mainly characterized by the proliferation of opportunistic pathogens and the depletion of commensal organisms [8,9,10]. While many investigations have largely focused on comparing the microbial compositions in COVID-19 patients with those in healthy controls [8,9], very few have explored how the COVID-19-associated microbiome changes differ from those observed in other respiratory diseases, such as H1N1 flu and pneumonia [8,11]. Furthermore, the existing studies, though informative, are limited by their small sample sizes (for example, the aforementioned studies used 6 pneumonia patient samples and 24 influenza patient samples, respectively). Evaluating the robustness of microbiome–disease associations requires large-scale comparisons across several case–control studies, often attained through meta-analyses. These meta-analyses aim to identify associations consistent across various studies, reducing the risk of attributing findings to biological confounders [12]. The majority of previous microbiome meta-analyses of COVID-19 patients have relied on 16S rRNA gene amplicon data and have revealed significant overall reductions in the microbiome diversity in COVID-19 patients, but these observations were affected by either low effect size or low resolution [13,14,15,16].

In contrast, shotgun metagenomics offers a higher taxonomic resolution, enhancing statistical power for identifying disease-associated microbes. Additionally, while microbial community shifts are observed in multiple diseases, elucidating unique aspects of microbial shifts in COVID-19 patients requires a cross-disease analysis involving various respiratory diseases with similar symptoms. Such analysis offers a comprehensive perspective on generalized and unique biomarkers associated with COVID-19. Moreover, real-world metagenomic data comprise millions of short reads that pose a significant challenge to alignment-based profilers. These reads harbor potential insights into microbial species shifts. Clearly, complementary strategies are needed for more robust metagenomic profiling. The incorporation of alignment-free methods, specifically those that are based on probabilistic model scoring, could provide a reliable classification at higher taxonomic ranks, as was demonstrated in previous studies [17]. To address the aforementioned challenges in COVID-19 microbiome analysis, we conducted a comprehensive meta-analysis incorporating six whole metagenomic shotgun sequencing studies focused on the gut and respiratory microbiomes. This comprehensive dataset comprised 429 disease-associated samples, including COVID-19-associated samples, and 215 control samples from both sources. Initially, we identified microbes exhibiting differential abundance between the COVID-19 and control groups. Subsequently, we employed machine learning to classify COVID-19 and control samples, thereby assessing the discriminatory potential of microbial abundance patterns. This analysis uncovered microbial features that were deemed important by machine learning models in distinguishing COVID-19 samples from control samples. Furthermore, to catalog both generalized and unique biomarkers associated with COVID-19, we compared the differentially abundant microbial biomarkers in COVID-19 samples with those in other respiratory diseases, including COPD, pneumonia, and pulmonary tuberculosis. We also conducted multiclass classification using machine learning algorithms by incorporating abundance data from COVID-19 samples and those from other respiratory diseases, including COPD, pneumonia, and pulmonary tuberculosis. The complementary strengths of both alignment-based (Kraken 2) and alignment-free (POSMM) methods were leveraged to characterize the microbiomes [17,18]. Our study not only highlights the potential diagnostic COVID-19 biomarkers but also underscores the efficacy of alignment-free methods in characterizing metagenomic reads left unclassified by alignment-based methods.

## 2. Materials and Methods

### 2.1. Metagenomic Data

Whole metagenome shotgun sequencing datasets for COVID-19 and other respiratory diseases, as well as healthy controls, spanning from 2020 to 2023 were collected from NCBI BioProject (www.ncbi.nlm.nih.gov/bioproject, accessed on 13 June 2023). These datasets were compiled from the gut and respiratory microbiomes. The gut microbiome datasets represented fecal samples collected from respiratory disease-afflicted individuals and healthy individuals. Respiratory microbiome samples were collected from nasopharyngeal swabs, pharyngeal swabs, and respiratory tracts. Our meta-analysis incorporated a total of 429 COVID-19 patients and 215 healthy controls, representing 3 gut microbiome cohorts and 3 respiratory microbiome cohorts from the USA, Japan, China, and Sweden (Table 1). Metagenome samples of pneumonia, COPD, and pulmonary tuberculosis were collected from the gut microbiome, with the exception of one dataset that represents the COPD respiratory microbiome (Table 1). Further details for all the datasets used in this study are provided in Supplementary File S1.

### 2.2. Taxonomic Classification

Prior to the analysis, the raw paired-end sequencing data underwent preprocessing, which entailed quality trimming, adaptor removal, and merging, using AdaptorRemovalV2 software (version 2.3.3) [19]. Subsequently, Kraken 2 was employed for the taxonomic classification of the reads, allowing us to establish a foundational understanding of the microbial composition of the samples [18].

To assess the relative abundance and distribution of taxa within the samples, Pavian was employed [20]. The relative abundance was calculated as the percentage of each taxon to the overall microbial community in the sample. Next, the reads in each sample that remained unclassified by Kraken 2 were classified using the alignment-free metagenomic profiling tool, POSMM [17]. This profiler uses a probabilistic scoring-based approach to classify the reads and provides a confidence score suitable for thresholding. All the unclassified reads were classified at the genus level above the confidence threshold of 0.25, which was reported to yield the highest sensitivity.

### 2.3. Biomarker Identification

**Figure 1:**
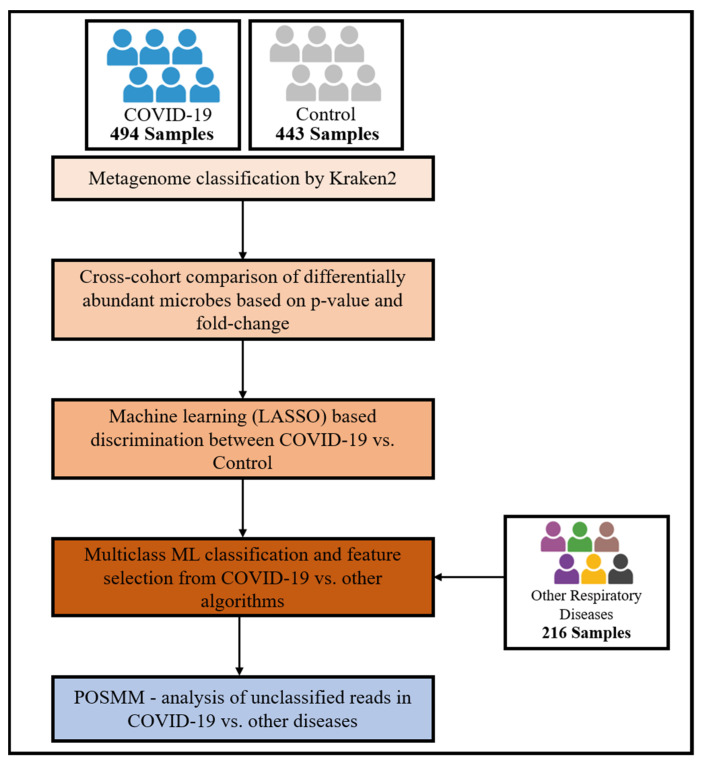
Workflow of the pipeline used for our COVID-19 meta-analysis for biomarker identification.

In this study, a statistical pipeline to decipher microbiome-based biomarkers associated with COVID-19 was used. As illustrated in Figure 1, the pipeline begins with the curation of whole genome shotgun sequencing samples representing microbiomes of COVID-19 and control cohorts sourced from diverse geographical locations. Next, the raw sequence data are subjected to preprocessing, followed by mapping and statistical analysis to derive abundance profiles of microbial taxa in the samples. These profiles are used to identify differentially abundant microbial taxa in COVID-19 subjects versus control subjects. The microbial abundance profiles are inputted into a machine learning classifier, allowing assessment of the discriminatory potential of these microbial features in distinguishing COVID-19 microbiome samples from control (healthy) samples. To identify microbial biomarkers specific to COVID-19 among the respiratory diseases with similar symptoms, we incorporated data from metagenomic studies of other respiratory diseases presenting symptoms similar to COVID-19, namely of COPD, pulmonary tuberculosis, and pneumonia. A multiclass machine learning classifier was trained on microbial abundance data from the aforementioned respiratory diseases, including COVID-19, and the respective healthy controls and then was tested on a held-out set for its ability to distinguish samples from different respiratory conditions. This approach also facilitated the identification of microbes that either exhibit broad involvement across multiple respiratory diseases or are uniquely associated with COVID-19. Finally, to characterize metagenomic reads left unclassified by the alignment-based profiler Kraken 2, we used POSMM, an alignment-free profiler. This allowed us to assess the trends of these candidate microbial biomarkers at higher taxonomic levels across the samples.

### 2.4. Differential Abundance Estimation for Classified Reads

**Figure 2:**
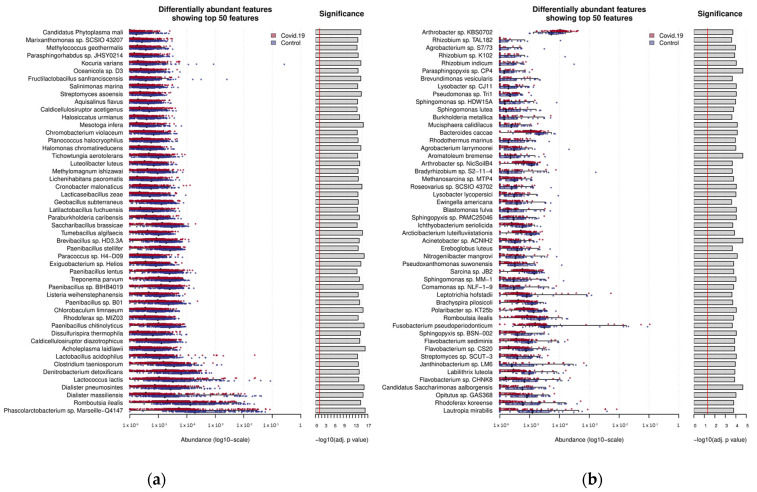
The abundance (boxplots) and their significance (negative logarithm of the *p*-value, based on the Wilcoxon test) of the top 50 differentially abundant species found in COVID-19 (**a**) gut microbiome and (**b**) respiratory microbiome datasets.

The SIAMCAT R package was used to filter out the features (microbes) with a relative abundance of less than 0.1%. The statistically significant differences in species abundances between the COVID-19 and control (healthy) microbiomes were estimated by the Wilcoxon test to determine differentially abundant taxa in COVID-19 samples. Further, the COVID-19 and control samples were grouped together based on their source, namely the gut and respiratory microbiomes. Following the statistical test, the top 50 differentially abundant microbial species in COVID-19 samples (versus control samples) from the gut and respiratory microbiomes were cataloged (Figure 2 and Supplementary Figure S1). This analysis was performed similarly for the other respiratory disease metagenome datasets, followed by a multi-disease comparison to identify differentially abundant microbes unique to COVID-19 and those that overlapped with differentially abundant microbes in other respiratory diseases.

### 2.5. Binary Machine Learning Analysis

The SIAMCAT package was used to train a machine learning model, namely LASSO regression, which employs a shrinkage and variable selection algorithm for linear regression [21]. This model was trained on relative abundance data of COVID-19 and control samples from the gut and respiratory microbiomes. Here, all samples in the respective gut and respiratory microbiome groups were used. The data from each group were divided into 80% for training the model and 20% for testing the trained model. The model was then tested for its ability to predict COVID-19 in an independent, held-out dataset. First, the features (relative abundance values) representing samples of both cohorts were log-transformed, and then the training and testing were performed using a 5-fold cross-validation procedure. A performance evaluation was conducted by generating an AU-ROC curve, which provides insights into the model’s discriminatory capacity. Next, the top discriminatory features selected by the machine learning model (sorted based on their median relative feature weight and robustness) were cataloged. Note that the feature weight of each microbe and its robustness were used as the criteria for selecting features of importance from all models, showing the weight proportion given to features included in over 50% of the cross-validation models using the SIAMCAT R package [22]. The relative feature weight served as an effect size measure, assigning higher weights to more relevant features, while robustness denoted the proportion of models that selected a particular feature [23]. Further, the Z-scores of top microbial features for COVID-19 versus control samples were examined for the deviation from each respective mean.

### 2.6. Multiclass Machine Learning Classification

We conducted a multiclass classification of COVID-19 and other respiratory disease samples and control samples. The other respiratory diseases considered here were pneumonia, COPD, and pulmonary tuberculosis (Table 1). To ensure consistency, the relative abundance estimation for these samples followed the same methodology applied for the COVID-19 samples. The relative abundance data for these diseases were incorporated with the COVID-19 data and normalized to serve as an input for the multiclass machine learning algorithm. For this analysis, we employed the XGBoost algorithm [24], which was implemented using the python “xgboost” module. XGBoost was chosen for its robust learning capabilities, especially in complex non-linear prediction tasks. Utilizing an ensemble of trees, XGBoost constructs a predictive model by iteratively improving upon previous iterations, while also incorporating built-in regularization techniques to mitigate overfitting risks. The dataset was divided into an 80% training set and a 20% testing set, and a 5-fold cross-validation was performed. The multi:softprob objective (loss) function was used as a learning parameter by XGBoost for the multiclass classification. The predictions were generated by calculating the probability that a sample belonged to a class, and the performance evaluation was conducted based on precision, recall (sensitivity), and F1 score using the Python “sklearn.metrics” module.

## 3. Results

### 3.1. A Meta-Analysis of COVID-19 and Other Respiratory Diseases’ Gut and Lung Microbiome Datasets

Our primary objective was to uncover microbial taxa that exhibit differential abundance in COVID-19 metagenomes compared to control samples. Initially, we performed the analysis of gut and respiratory metagenomic datasets individually using Kraken 2 (version 2.1.3) [18]. Following the identification of microbial taxa represented in each sample, the relative abundance was quantified using Pavian (version 1.0) by comparing all samples in each dataset. The classified portion of the metagenomes enabled the identification of species with significant differential abundance within the individual datasets as well as the grouped gut microbiome and respiratory microbiome datasets (relative to the control). The complete list of significantly abundant species represented in these datasets are provided in Supplementary File S2. Our analysis revealed both unique and shared differentially abundant species in the datasets (*p*-value < 0.05). The top 50 species exhibiting differential abundance in each of the gut and respiratory microbiomes, ranked by their *p*-values, are shown in Figure 2. The majority of these differentially abundant species were underrepresented in COVID-19 samples compared to control (healthy) samples, in concurrence with the previous studies [13,15]. In the three COVID-19 gut microbiome datasets, a significant reduction in several beneficial bacteria was observed. Among the most depleted beneficial bacteria in COVID-19 gut microbiome samples were from the genus *Dialister*, including *Dialister massiliensis* (*p*-value: 3.73 × 10^−16^) and *Dialister pneumosintes* (*p*-value: 1.74 × 10^−16^), from the order *Lactobacillales*, including *Lactobacillus acidophilus* (*p*-value: 2.35 × 10^−14^), *Lactococcus lactis* (*p*-value: 1.03 × 10^−14^), *Latilactobacillus fuchuensis* (*p*-value: 2.29 × 10^−14^), *Fructilactobacillus sanfranciscensis* (*p*-value: 2.17 × 10^−15^), and *Lacticaseibacillus zeae* (*p*-value: 1.71 × 10^−14^), and from the order *Bacillales*, including multiple species from the genus *Paenibacillus* (*p*-value: 1.04 × 10^−13^), *Brevibacillus* sp. HD3.3A (*p*-value: 7.02 × 10^−15^), and *Paracoccus* sp. H4-D09 (*p*-value: 1.58 × 10^−16^) (Figure 2a). The abundance levels of these species were also previously reported to decrease in COVID-19 patients [15,25,26]. All of these species are known to be predominantly present in the healthy gut microbiome. Additionally, novel microbes that were not previously reported to be affected by COVID-19 were uncovered, including *Phascolarctobacterium* sp. Marseille-Q4147 (*p*-value: 7.95 × 10^−17^), *Romboutsia ilealis* (*p*-value: 2.36 × 10^−15^), *Kocuria varians* (*p*-value: 2.00 × 10^−15^), and *Acholeplasma laidlawii* (*p*-value: 8.10 × 10^−17^). These bacteria are also known to be nonpathogenic, but they were not previously reported to be affected in COVID-19 patients [27,28,29,30]. In contrast, we found only 35 species that were found to be significantly overrepresented in COVID-19 gut microbiome samples compared to the control samples. Among the top overabundant bacteria in COVID-19 gut microbiome samples were those from the genus *Bacteroides*, for example, *Bacteroides fragilis* (*p*-value: 2.88 × 10^−5^), *Bacteroides luhongzhouii* (*p*-value: 0.00038), and others. While these are typically commensal bacteria, certain conditions can lead to their overgrowth or dysregulation, which may contribute to disease states. Studies have shown that *Bacteroides* species can play a role in exacerbating inflammation in these conditions by producing pro-inflammatory molecules or triggering immune responses [31]. Inflammatory diseases such as Crohn’s disease and ulcerative colitis are characterized by chronic inflammation in the gut [32,33]. Other commonly known opportunistic pathogens such as *Parabacteroides distasonis* (*p*-value: 1.22 × 10^−11^), *Staphylococcus aureus* (*p*-value: 0.002), *Paracoccus mutanolyticus* (*p*-value: 0.002), *Myroides odoratimimus* (*p*-value: 0.004), and *Phocaeicola vulgatus* (*p*-value: 0.000615281) were found enriched in the COVID-19 gut microbiome. Many of these bacteria play a dichotomous role in a variety of diseases such as inflammatory bowel disease (e.g., Crohn’s disease and ulcerative colitis), diabetes, and several autoimmune diseases [34]. Previous studies have shown that some of these bacteria promote intestinal inflammation [35,36]. Inflammation is the regular response of the body against pathogens, but it is observed at higher levels in COVID-19 patients [35]. Uncontrolled inflammation can lead to a cytokine storm (this is caused by the loss of regulation of pro-inflammatory cytokines such as IL-1 and IL-6, which leads to the release of a large number of cytokines) [36]. A higher level of cytokines has a higher risk of causing multiple organ failure. This had been observed in some of the COVID-19 patients and could be attributed, in part, to microbial dysbiosis [37].

For the respiratory COVID-19 microbiome data, the overall microbial diversity was found to be much lower than that in the COVID-19 gut microbiome (Figure 2b). Species from the genus *Flavobacterium*, including *Flavobacterium* sp. CHNK8 (*p*-value: 0.00013) and *Flavobacterium* sp. CS20 (*p*-value: 0.00013), and the genus *Lysobacter*, including *Lysobacter* sp. CJ11 (*p*-value: 9.02 × 10^−5^) and *Lysobacter lycopersici* (*p*-value: 9.68 × 10^−5^), were among the novel bacterial species underrepresented in the COVID-19 microbiomes. Apart from them, some bacteria previously reported to be differentially abundant were also identified [16]. These belong to the genus *Prevotella*, including *Prevotella jejuni (p*-value: 0.0013) and *Prevotella copri* (*p*-value: 0.003), the genus *Roseburia*, including *Roseburia* sp. NSJ-69 (*p*-value: 0.0044) and *Roseburia intestinalis* (*p*-value: 0.005), the genus *Bacteroides*, and the family *Sphingomonadaceae*. Many of these are known to be beneficial bacteria and were found to be underrepresented in COVID-19 patients. *Prevotella* species are major components of the endogenous airway microbiome and play a role in reducing bacterial infections from multiple pathogens [38,39]. Multiple biomolecules from these species have been reported to be involved in their antimicrobial properties, including enzymes and secondary metabolites, demonstrating their activity against a range of pathogenic microorganisms [40]. Butyrate-producing bacteria such as *Roseburia* help in regulating immunity and in maintaining epithelial barrier integrity through interleukin-22, thus promoting immune system tolerance [41,42]. On the other hand, among the top 50 differentially abundant species, only *Arthrobacter* sp. KBS0702 (*p*-value: 0.000189) was found to be overabundant in the COVID-19 nasopharyngeal microbiome. Others microbes significantly over-abundant in the COVID-19 nasopharyngeal microbiome include those from the families *Legionellaceae* (*L. pneumophila*), *Kitasatosporales* (*Streptomyces* sp. GMY02 and *S. olivaceus*), and *Burkholderiaceae* (*P. caribensis* and *R. solanacearum*). All of these are known to be opportunities pathogens. Of these, *L. pneumophila* is a major causative agent of severe pneumonia (lung infection) and community-acquired pneumonia (CAP) and has also been reported to cause the co-infection with COVID-19 and can be potentially fatal [43,44]. *Streptomyces* and *P. caribensis* are associated with pathogenic agents in multiple respiratory infections [45].

### 3.2. Classification of COVID-19 and Control Microbiome Samples Based on Microbial Abundance Using Machine Learning

**Figure 3:**
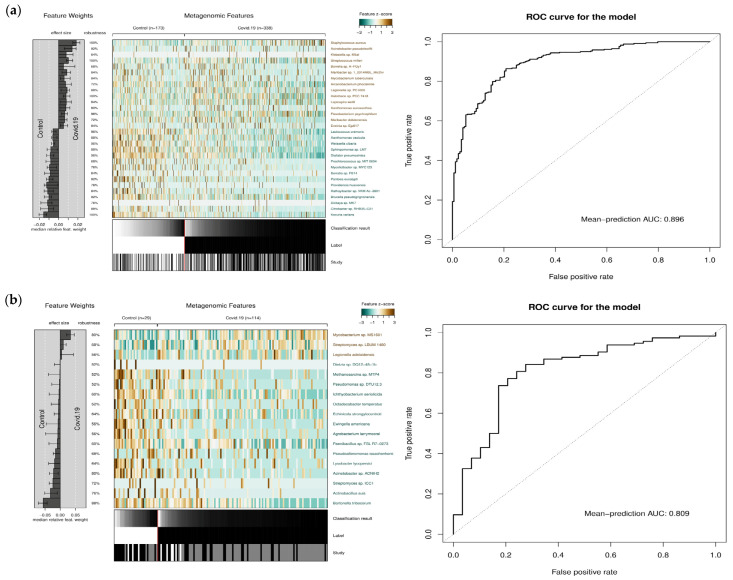
Heatmaps for metagenomic features deemed important by the machine learning classifier and their effect size, robustness, and z-score, as well as the ROC curve for classification, are shown for (**a**) gut microbiome and (**b**) nasopharyngeal microbiome COVID datasets.

Because of the differential abundance of microbes in the COVID-19 versus control samples, we posited that microbial abundance can be used as a statistical feature in discriminating COVID-19 samples from control samples or in the diagnosis of COVID-19. To assess the predictive capability of microbiome abundance data for distinguishing COVID-19 patient samples from healthy controls, we utilized a machine learning model, LASSO. This model also allowed identifying discriminatory features in COVID-19 versus control samples within both the gut and respiratory microbiome datasets in an unbiased way, in contrast to the statistical analysis described in the previous section, where the significance of microbial abundance was assessed for each microbe separately. Machine learning models provide an integrated framework that can decipher complex patterns or relationships that may not be apparent with standard statistical analyses. Here, we also assessed the overlap of a set of microbial species that were deemed discriminatory by the machine learning model with the set of differentially abundant species inferred using the statistical analysis. The accuracy of the machine learning model in discriminating the COVID-19 microbiome samples from the control samples, using the accuracy metric AU-ROC (area under receiver operating characteristic curve) was 0.90 for the gut microbiome and 0.80 for the nasopharyngeal microbiome (Figure 3a,b), demonstrating the ability of the model to discriminate COVID-19 samples from control samples based on microbiome features. With the model performing reasonably well in discerning different sample types, we proceeded to examine the key discriminatory features identified by the model (Figure 3). These microbial features were selected based on their relative feature weight and robustness [23].

Of the top 30 gut microbiome features (species) selected by the model, ~90% overlapped with the set of differentially abundant species inferred based on the statistical analysis described in the previous section. The majority of them belong to the phyla *Gammaproteobacteria, Actinomycetota*, and *Bacillota*. These include *Kocuria varians* (*p*-value: 2.00 × 10^−15^), *Rathayibacter* sp. VKM Ac-2801 (*p*-value: 4.54 × 10^−10^), *Mycolicibacter* sp. MYC123 (*p*-value: 2.73 × 10^−8^), *Xanthomonas vasicola* (*p*-value: 2.80 × 10^−8^), *Lactococcus cremoris* (*p*-value: 1.61 × 10^−13^), *Weissella cibaria* (*p*-value: 2.13 × 10^−12^), *Citrobacter* sp. RHB35-C21 (*p*-value: 5.16 × 10^−9^), *Dialister pneumosintes* (*p*-value: 1.74 × 10^−16^), *Pantoea eucalypti* (*p*-value: 2.05 × 10^−11^), and others. While the aforementioned species were found underrepresented in COVID-19 microbiome samples, those found overrepresented include *Staphylococcus aureus* (*p*-value: 0.0019), *Maribacter dokdonensis* (*p*-value: 0.00134), *Borrelia* sp. A-FGy1 (*p*-value: 0.0094), and *Arcanobacterium phocisimile* (*p*-value: 0.0243).

Similarly, of the top-ranked microbial species in the nasopharyngeal microbiome, only 18 species were selected based on their feature weight. Although only 6 of these species are represented in the list of the top 50 differentially abundant microbes, 10 of the remaining 12 species were also significantly differentially abundant. These six microbes were *Acinetobacter* sp. ACNIH2 (*p*-value: 2.15 × 10^−5^), *Lysobacter lycopersici* (*p*-value: 9.68 × 10^−5^), *Agrobacterium larrymoorei* (*p*-value: 0.0001), *Methanosarcina* sp. MTP4 (*p*-value: 0.0002), *Ichthyobacterium seriolicida* (*p*-value: 0.0002), and *Ewingella americana* (*p*-value: 0.0002). All of these were underrepresented in COVID-19 microbiome samples.

### 3.3. Multi-Disease Comparison of Differentially Associated Microbes

**Figure 4:**
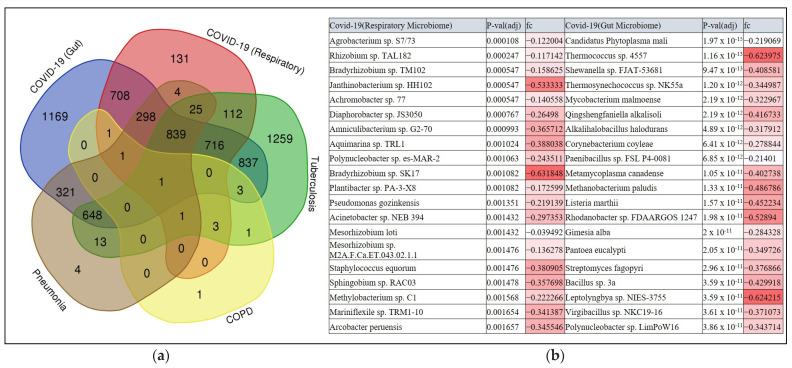
(**a**) A Venn diagram showing a multi-disease comparison of differentially abundant species overlap. (**b**) Top 20 species uniquely identified to be differentially abundant in COVID-19 patients (in the gut and respiratory microbiomes) based on *p*-value significance.

While the binary classification described in the previous section highlighted the microbial features deemed important for discriminating COVID-19 samples from healthy samples, such features may also be shared with other respiratory diseases. Here, we conducted metagenomic profiling for other respiratory diseases exhibiting symptoms akin to COVID-19. These were COPD, pneumonia, and pulmonary tuberculosis, sourced from independent studies (Table 1). We then compared the differential abundance of microbes in the individual diseased subjects (relative to their respective controls used in each study). Further, the significantly differentially abundant microbes obtained in individual studies were compared with those obtained for COVID-19 studies (Supplementary File S3). This analysis led to the identification of differentially abundant bacteria unique to COVID-19 (Figure 4). Overall, 1169 and 131 bacterial species differentially abundant in COVID-19 gut and respiratory microbiome samples, respectively, were found to be uniquely associated with COVID-19 (Supplementary File S4). Among these were species belonging to the group harboring xenobiotic metabolizing enzyme repertoire, encompassing the genera *Bradyrhizobium*, *Rhizobium*, *Methylobacterium*, *Neisseria*, and *Bacillus* in the respiratory microbiome [46,47]. Bacterial genera known for producing specialized metabolites (some of them also known for having biosynthetic gene clusters (BGCs)), including *Corynebacterium*, Paenibacillus, Prevotella, Citrobacter, and Burkholderia, were observed to be uniquely associated with the COVID-19 gut microbiome [47,48]. Further, we identified multiple shared species that were depleted and are known to utilize tryptophan and produce indole-3-aldehyde, subsequently inducing IL-22 production by innate lymphoid cells (ILCs), such as *Lactobacillus*, *Clostridium*, and *Bacteroides* sp. [47]. The reduction in the abundance of these microbial species may stem from heightened inflammation in COVID-19 patients, which impacts the metabolic activities of host microbes involved in defense mechanisms such as xenobiotic detoxification.

### 3.4. Multiclass Machine Learning Classification

**Figure 5:**
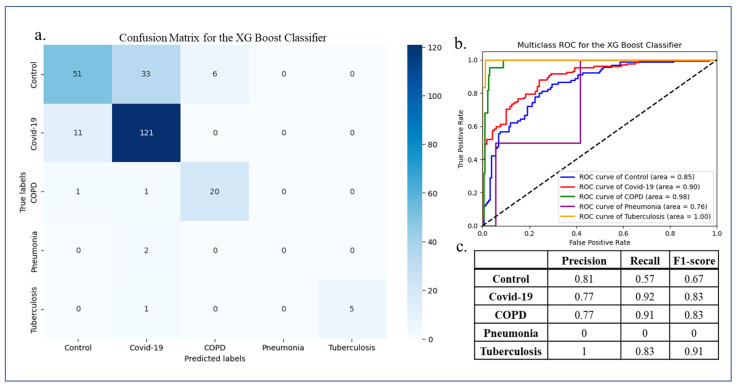
Multiclass classification and performance evaluation of 5 sample types: control, COVID-19, pneumonia, COPD, and pulmonary tuberculosis. (**a**) Confusion matrix for the prediction; (**b**) multiclass ROC curve; and (**c**) precision, recall, and F1 score for each sample type.

In our next analysis, we used a multiclass classification approach to further discern the COVID-19 microbiome by integrating metagenomic data from other respiratory diseases sharing similar symptoms. This was aimed at deciphering both the generalized biomarkers shared across the respiratory disease microbiomes and the unique biomarkers characteristic of the COVID-19 microbiome. The other respiratory diseases included COPD, pulmonary tuberculosis, and pneumonia. Their metagenomic data were collated with the COVID-19 disease datasets and the microbial relative abundance data were obtained for each disease. Here, we used a machine learning algorithm, XGBoost, for the classification of microbiomes representing control, COVID-19, COPD, pneumonia, and pulmonary tuberculosis patients, with 953 samples in total. Known for its ensemble of decision trees and built-in regularization techniques, XGBoost offers robust learning capabilities, mitigating the risks of overfitting and facilitating a holistic understanding of the data. Evaluation metrics including precision, recall, and F1 score were used as before to assess the performance. XGBoost yielded an F1 score of 0.83 and an AU-ROC value of 0.90 for the discrimination of COVID-19 microbiome samples from the other respiratory disease and control samples (Figure 5). True and false predictions for each sample type in the test data are displayed in the confusion matrix (Figure 5a). We also assessed the overlap of COVID-19 microbial biomarkers deciphered based on the statistical analysis (Section 3.1) with the microbial features that were deemed important in discriminating COVID-19 microbiome samples from other respiratory disease microbiome and control samples. Out of the microbial features deemed important for multiclass classification for discriminating all diseased conditions, we found that 189 of the unique COVID-19 microbiome biomarkers (10%) were also identified as discriminatory COVID-19 features by XGBoost (Supplementary File S5).

The microbes uniquely associated with COVID-19 that were also deemed important for discrimination in this classification were cataloged as high-confidence COVID-19 biomarkers. These were from the genera *Phascolarctobacterium*, *Prevotella*, *Paenibacillus*, *Neisseria*, *Citrobacter*, *Burkholderia*, *Bacillus*, *Lactobacillus*, *Streptomyces*, *Streptococcus*, *Dialister*, *Ralstonia*, *Weissella*, *Bifidobacterium*, and *Salmonella*.

Microbes that were uniquely associated with the COVID-19 samples but were not deemed important by the machine learning classifier included those belonging to the genera *Xanthomonas*, *Sphingomonas*, *Ruminococcus*, *Paracoccus*, and *Mycolicibacterium*.

### 3.5. Classifying the “Unclassified” Microbiome Reads Using POSMM

**Figure 6:**
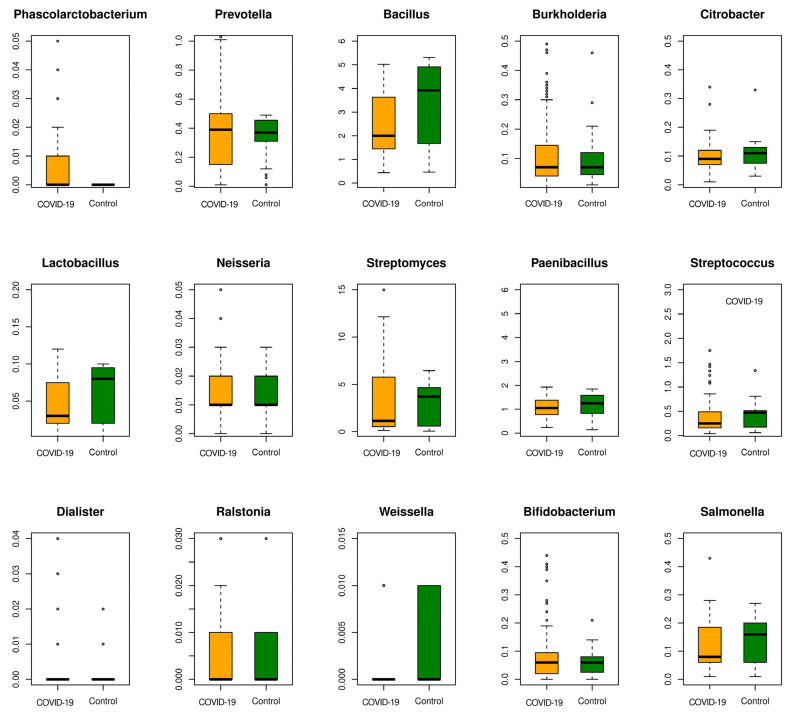
A boxplot showing the abundance of genera in the COVID-19 and control samples based on POSMM’s predictions.

Kraken 2 is among the frequently used composition-based profilers, providing high-precision classification of metagenomic reads. It is based on the *k*-mer-based exact matches of the reads to the genomes in the database. However, if the *k*-mers extracted from these reads could not be matched to the genomes of their originating species, apparently due to the absence of these genomes in the database, those reads are left unclassified. In analyzing complex real-world metagenomic data, we obtained a range of unclassified read percentages for the datasets (Supplementary Figure S2). For the analysis of unclassified reads from Kraken 2, we used the alignment-free probabilistic scoring method, POSMM. This enables the classification of these reads, though at the higher taxonomic levels. This analysis was performed for the COVID-19 microbiome and the respective control samples. After the taxonomic classification of each of these reads by POSMM, the percentage of reads representing each taxon was re-estimated for each sample. The differential abundance of microbes in the COVID-19 microbiome was re-estimated at the genus level. The most differentially abundant genera represented in the COVID-19 microbiome samples were *Phascolarctobacterium*, *Prevotella*, *Paenibacillus*, *Neisseria*, *Citrobacter*, *Burkholderia*, *Bacillus*, *Lactobacillus*, *Streptomyces*, *Streptococcus*, *Dialister*, *Ralstonia*, *Weissella*, *Bifidobacterium*, and *Salmonella* (Figure 6). Many of these microbial groups were also reported in previous COVID-19 studies, validating POSMM’s capacity to uncover microbial taxa represented in unclassified reads.

The genera *Bacillus*, *Lactobacillus*, *Prevotella*, *Streptomyces*, *Paenibacillus*, *Weissella*, *Streptococcus*, and *Salmonella* were found underrepresented in the COVID-19 samples. In contrast, genera such as *Citrobacter*, *Burkholderia*, and *Bifidobacterium* were not found differentially abundant in COVID-19 microbiome samples (versus the control samples), although several species from these genera were found differentially abundant. This suggests that these genera consist of species, some of which are differentially abundant while others are not.

## 4. Discussion

In this study, we examined the gut and respiratory microbiota associated with COVID-19 patients and healthy individuals, as well as the microbiota from other respiratory diseases, and identified microbial biomarkers that are unique to COVID-19 and those that are conserved across respiratory diseases. These findings shed a light on the intricate interplay between the human microbiome and COVID-19, providing new insights into the microbial dysbiosis associated with this viral infection. Through comprehensive analyses of the gut and respiratory metagenomic datasets, we discerned a marked depletion of certain bacteria in COVID-19 patients compared to healthy controls, corroborating previous observations in the literature [15,25,26]. Notably, microbes belonging to the genera *Bacillus*, *Lactobacillus*, *Prevotella*, *Paenibacillus*, *Weissella*, *Streptococcus*, and *Salmonella* exhibited significantly lower abundance levels in COVID-19 samples, underlining the potential role of some of these bacteria in maintaining gut homeostasis and immune function [38,39,46,49]. These microbes were deciphered using both the classical statistical approach and the machine learning approach (binary and multiclass). This difference in abundance was also reflected in the unclassified portion of samples when they were later characterized using POSMM. Conversely, we identified an overabundance of certain opportunistic pathogens, including *L. pneumophila*, *Streptomyces*, and *P. distasonis*, in COVID-19 patients, implicating them in disease pathogenesis [34,43,44,45]. The observed dysregulation of microbial communities, particularly in the gut, aligns with prior studies linking alterations in the microbiome to inflammatory conditions and immune dysfunction, thus providing valuable insights into the pathophysiology of COVID-19.

Our multiclass classification analysis, incorporating data from other respiratory diseases, provided valuable insights into the specificity of microbial shifts associated with COVID-19. By integrating metagenomic information from the COVID-19, COPD, pulmonary tuberculosis, and pneumonia datasets, we elucidated the power of machine learning in discriminating different respiratory disease types based on microbial abundance. Our analysis also revealed distinct microbial signatures associated with COVID-19, highlighting the potential of microbiome profiling in disease stratification and differential diagnosis (Figure 4 and Figure 5).

Additionally, our study leveraged the alignment-free metagenomic profiler, POSMM, to gain further insights into the microbial dysbiosis in COVID-19. By classifying the reads left unclassified by Kraken 2, POSMM elevated the level of sensitivity in metagenomic profiling, rendering a rather complete profiling of a microbiome. The integration of POSMM into our analytical pipeline proffered a more comprehensive and nuanced understanding of the microbial communities implicated in disease pathogenesis.

Despite the new insights gained through this study, several limitations should be acknowledged. First, publicly available metagenomic data were used in this analysis, which may vary in their sample collection methods, sequencing technologies, and the data processing pipelines. This heterogeneity could add biases and confounders that may affect the generalizability of our findings. Additionally, this study can be expanded in the future with larger sample sizes to improve the statistical power and robustness of the analysis. Future studies that could integrate functional omics approaches, such as metatranscriptomics and metabolomics, could provide deeper insights into the metabolic activities and functional pathways of the microbial communities in COVID-19 patients.

## 5. Conclusions

This study presented a computational pipeline for the meta-analysis-based detection of candidate microbes specific to COVID-19 patients, demonstrating the potential use of these microbes as distinctive signatures for COVID-19 diagnosis. Through statistical and machine learning analyses, we detected significant microbial shifts in the COVID-19 microbiomes from the microbiomes of healthy controls and of patients with other respiratory diseases. This approach addressed the knowledge gap by deciphering COVID-19 and other respiratory disease-specific microbial alterations that enhanced our understanding of the impact of COVID-19 on the gut and lung microbiomes.

## Figures and Tables

**Table 1:** Metagenomic datasets used in the analysis.

Discovery Datasets		BioProject ID	Number of Samples	Number of Diseased Samples	Number of Control Samples	Source	Year	Country
COVID-19	1	PRJDB13214	208	103	105	Gut microbiome	2023	Japan
	2	PRJNA781460	57	39	19	Respiratory microbiome	2021	Sweden
	3	PRJNA656660	9	6	3	Respiratory microbiome	2020	China
	4	PRJNA743981	99	79	20	Respiratory microbiome	2021	USA
	5	PRJNA624223	50	15	15	Gut microbiome	2020	China
	6	PRJNA650244	240	187	53	Gut microbiome	2020	China
Other respiratory diseases								
Pneumonia	1	PRJNA624223	21	6	15	Gut microbiome	2020	China
COPD	2	PRJNA562766	57	29	28	Gut microbiome	2020	Australia
COPD	3	PRJNA852674	135	99	36	Respiratory microbiome	2022	China
Pulmonary tuberculosis	4	PRJNA401385	77	46	31	Gut microbiome	2017	China

## Data Availability

Data is contained within the article and Supplementary Material.

## Supplementary Materials

The following supporting information can be downloaded at: https://www.mdpi.com/article/10.3390/microorganisms12061058/s1, Figure S1: Heatmap showing the fold change in the individual COVID-19 studies comparing the top abundant microbes in (a) gut microbiome and (b) nasopharyngeal microbiome datasets, * indicates the fold change with *p*-value < 0.05; Figure S2: Representation of the percentage of unclassified reads across multiple datasets; File S1: Details of all the samples in the COVID-19 studies; File S2: List of species significantly abundant in the COVID-19 gutand nasopharyngeal microbiome datasets; File S3: List of species significantly abundant in COPD, pneumonia, and pulmonary tuberculosis; File S4: List of common and unique species present in multiple samples based on a Venn diagram; File S5: Species that overlapped are uniquely associated with COVID-19 and multiclass ML features.
