# Supplementary figures and images for: Deciphering Microbial Shifts in the Gut and Lung Microbiomes of COVID-19 Patients
Source: Microorganisms. 2024 May 24;12(6):1058. doi: 10.3390/microorganisms12061058 (PMC11205787; doi:10.3390/microorganisms12061058)

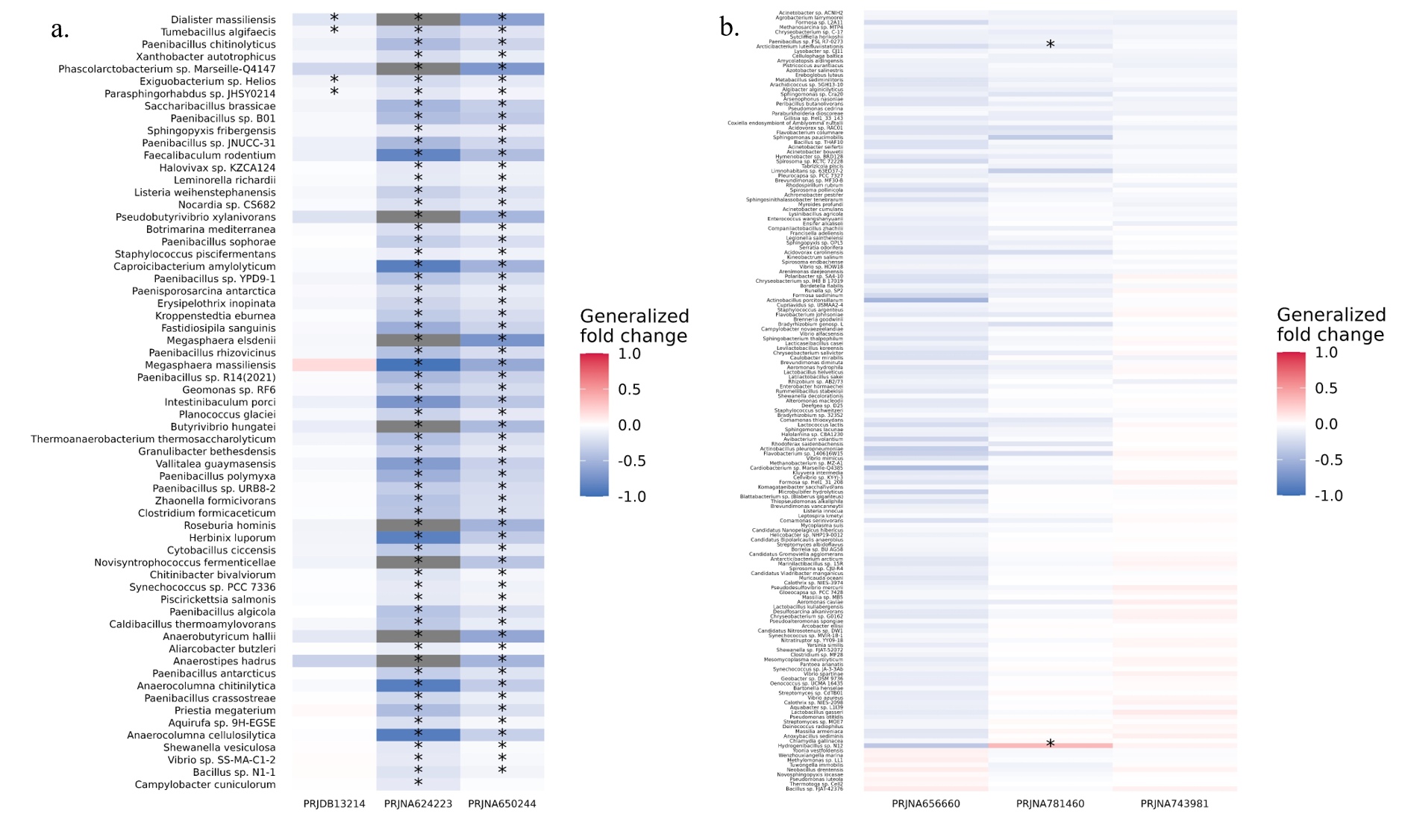

Supplement: Supplementary file 1 [file microorganisms-12-01058-s001.zip › Supplementary Figure S1.png]

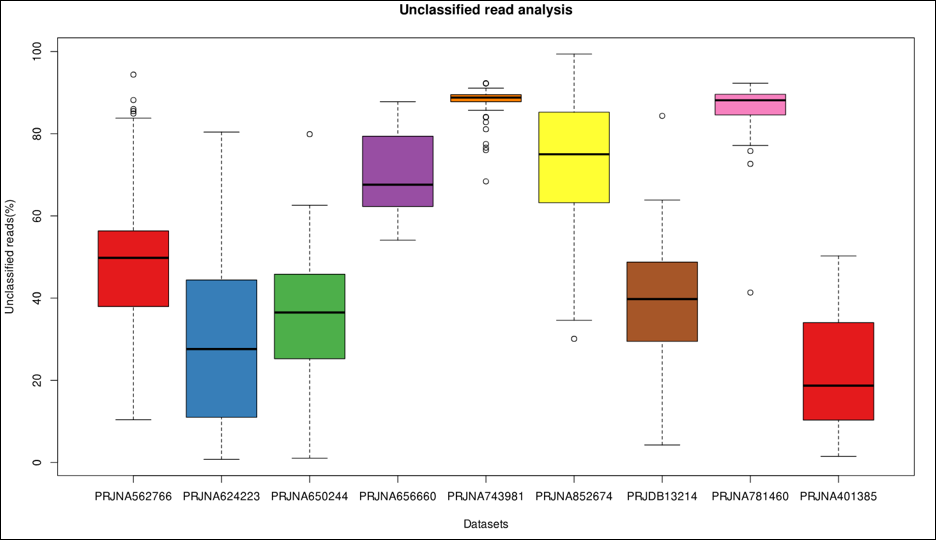

Supplement: Supplementary file 1 [file microorganisms-12-01058-s001.zip › Supplementary Figure S2.png]
